# Sexology and development

**DOI:** 10.1177/09526951231213970

**Published:** 2023-12-06

**Authors:** Chiara Beccalossi, Kate Fisher, Jana Funke

**Affiliations:** 4547 University of Lincoln, UK; 151763 University of Exeter, UK; 151763 University of Exeter, UK

**Keywords:** development, sexology, sexual science, sexuality, transnational

## Abstract

The history of sexology is a well-established field of scholarly investigation animated by ongoing contestations around the disciplinary boundaries, political outlook, and transnational dimensions of the sexological field. This special issue focuses on the multivalent concept of development to address some of the most pressing questions driving current historiographical conversations in this area. The five articles examine how sexology developed in the late 19th and 20th centuries and explore how sexologists deployed various developmental categories to understand sexuality in different national, geographical, and linguistic spaces, including India, Latin America, and Western and Southern Europe. They show how central tracing the relationship between sexuality and human development became to sexologists’ understanding of their project and its value. By interrogating the intersecting individual, social, cultural, and evolutionary developmental frameworks at the heart of sexological knowledge production, the articles engage with sexology as a global and transnational project deeply shaped by ideologies of race, nation, and empire and motivated by a diverse range of political concerns and intellectual questions. In so doing, the special issue as a whole demonstrates the breadth of the sexological field in terms of its interdisciplinary scope, diverse political and intellectual agendas, and global dimensions.

Over half a century after Michel Foucault's foundational exploration of *scientia sexualis*, the history of sexology is a well-established area of scholarly investigation animated by ongoing contestations around the disciplinary boundaries, political outlook, and transnational dimensions of the sexological field (Bauer, 2015; Haynes, Fuechtner, and Jones, 2018; Giami and Levinson, 2021; Kahan and LaFleur, 2023; Leng and Sutton, 2021). This special issue focuses on the multivalent concept of development to address some of the most pressing questions driving current historiographical conversations in this area. The five articles examine how sexology developed in the late 19th and 20th centuries and explore how sexologists deployed concepts of development in different national, geographical, and linguistic spaces, including India, Latin America, and Western and Southern Europe. The authors brought into dialogue here interrogate different meanings of development to engage with sexology as a global and transnational project deeply shaped by ideologies of race, nation, and empire and motivated by a diverse range of political concerns and intellectual questions.

One of the most recognizable forms of evidence used in foundational sexological publications, such as Richard von Krafft-Ebing's *Psychopathia Sexualis* (1886) or Havelock Ellis and John Addington Symonds’s *Sexual Inversion* (1897), was the sexological case study (Damousi, Lang, and Sutton, 2015; Oosterhuis, 2000). Written in the first or third person, case studies offered (often highly detailed) narratives of individual sexual development over the life course. The case study allowed sexologists to chart and discuss how and why the sexed body, sexual desire, and gender identity developed. One of the most widely debated questions in early sexology and adjacent fields, including psychoanalysis, was whether sexuality was inborn and unchanging or shaped by external influences. Reform-oriented sexologists such as Ellis and Symonds in Britain and Magnus Hirschfeld in Germany, who have received a significant amount of attention in existing scholarship (Bauer, 2017; Crozier, 2008; Marhoefer, 2022; Nottingham, 1999), tended to present sexual orientation as congenital or inborn. This argument offered a powerful means to oppose homophobic accusations about so-called corruption and seduction and allowed sexologists to counter the moral condemnation and criminalization of same-sex desire. Yet congenital models were not the only frameworks available to sexual scientists. German sexologists, including Iwan Bloch and Albert Moll or the Austrian founder of psychoanalysis, Sigmund Freud, were eager to acknowledge other causative factors, including family dynamics or exposure to ‘obscene’ literature and art (Kahan, 2019). Moving beyond Northern and Western Europe demonstrates further that sexologists developed multiple frameworks for understanding the causes and origins of sexual desire. As Ryan Jones's article in this special issue demonstrates, for instance, Mexican sexology in the first decades of the 20th century tended to view homosexuality as caused largely by social and environmental rather than congenital factors.

Acknowledging that the sexual instinct was not static or fixed, but potentially pliable, meant that the individual's sexual development could be influenced or modified through biological, psychological, or behavioural interventions. Sexologists considered how various technologies, including hypnotic treatments and endocrinological interventions, could be employed to try to alter or guide the process of individual development. In Germany, sexologists such as Moll or Albert von Schrenck-Notzing explored the uses of hypnosis to try to alter same-sex attraction and reduce other apparently ‘undesirable’ sexual behaviours, including masturbation (Moll, 1921; von Schrenck-Notzing, 1895). As Chiara Beccalossi's article shows in relation to Southern Europe and Latin America, biotypological research cut across the fields of sexology, eugenics, and endocrinology, and often served to regulate bodies and desires that did not conform to normative standards of masculinity and femininity through hormonal treatments. Sexology was not simply concerned with labelling and classifying existing forms of gender and sexual nonconformity, but also authorized itself as a field capable of controlling and potentially changing individual sexual development. As this special issue demonstrates, these efforts served eugenic, nationalist, and imperialist ideologies, with sexologists legitimating their work by promising to create what they perceived as a stronger, fitter, or more civilized population.

Individual development, then, was inextricably intertwined with questions of social, cultural, and evolutionary development. This is evident in 19th-century evolutionary theories of so-called recapitulation, famously (and controversially) articulated by the German zoologist Ernst Haeckel, which suggested that individual development (ontogeny) repeated the development of the species (phylogeny). Hirschfeld, for instance, positioned himself as Haeckel's intellectual heir: both his study of *Die Gesetze der Liebe* (The Laws of Love; Hirschfeld, 1912) and a lecture theatre at his Berlin Institute of Sexology were dedicated to Haeckel. According to Hirschfeld, Haeckel's claims about the sexually undifferentiated origins of remote evolutionary ancestors, which were mirrored by the human individual in the early stages of embryonic development, provided vital evidence of the natural basis of homosexuality and other expressions of sexual and gender nonconformity (Hirschfeld, 1914: 635). This evolutionary account also had less affirmative implications in associating queer sexualities, including homosexuality, with ‘primitive’ and ‘backward’ stages of individual and evolutionary development. Freud believed that sexual nonconformity was often due to individuals being ‘held back’ in their sexual development (2001[1901–5]: 227). Similarly, Ellis associated homosexuality with ‘arrested development’ and a ‘primitive ancestral phase’ (1915: 256, 313). These entanglements of individual and evolutionary timelines created complex relationships between constructions of gendered, sexual, racial, and cultural differences (Rohy, 2009), which several contributions in this special issue interrogate.

While sexology has often been understood as a field concerned primarily with the development of so-called sexual perversions and pathologies, its remit was wider. As different authors in this special issue reveal, sexologists also considered ‘healthy’ and ‘normal’ bodies and desires, including by investigating heterosexuality and reproduction. In addition, sexologists explored the sexual manifestations of groups, or saw individuals as representatives of broader cultures, populations, or ‘races’. The association between queer sexualities and supposedly primitive stages of development racialized distinctions between the morbid, the perverse, and the healthy. The identification of sexual pathologies was refracted through racialized concerns about human development and the racial health of Western populations. European sexologists, such as Krafft-Ebing, Ellis, or Moll, rarely mentioned the racial identities of their subjects explicitly, yet, as scholars of American sexology have shown, white self-definition was amplified by connecting sexual and gender nonconformity with Blackness (Snorton, 2017; Somerville, 2000; Stein, 2015; Stone, 2023; Terry, 1999). As has long been understood, sexology (for instance in France) was intertwined with eugenic concerns about degeneration, disease, and national fitness, fuelled by anxieties that prophesied European civilizational decline and imperial collapse (Nye, 1991; Schaffner, 2012). At stake in the observation and analysis of individuals explored via case studies (and other methods) was the developmental status of imperial nations, described as belonging to ‘higher’ ‘Western’ or ‘civilized races’, and distinct from those ‘races’ labelled as ‘lower’, ‘primitive’, ‘savage’, ‘heathen’, ‘barbarous’, ‘polygamous’, or ‘Eastern’, to borrow the terms found in the works of Bloch, Krafft-Ebing, and Ellis. Although, as Heike Bauer has shown in the case of Hirschfeld, some sexologists were highly critical of biologized racial categories, and argued that differences between peoples reflected cultural variation (Bauer, 2010), a racialized framework for understanding the world in terms of developmental differences ensured that sexology remained implicated, indeed invested, in imperial politics of racial progress.

The complex relationship between imperial politics and sexual science is explored by several articles in this special issue. Many sexologists, such as Bloch and Hirschfeld in Germany and Paolo Mantagazza in Italy, deployed developmental categories to understand sexual customs around the globe. In so doing, they purposefully contributed material of relevance, in an imperially structured world, to the practice of colonial governance and regulation (Willey, 2016). Sexological data served multiple imperial functions: to justify colonial practices, to explain geopolitical power, to stoke fears of civilizational decline, or to puncture presumptions of imperial superiority and natural domination. As Alison M. Downham Moore's article shows, Muslim cultures were often constructed as ‘backward’. The oppression of women was a particularly frequent theme, with sexologists such as Krafft-Ebing arguing that it was ‘primitive’ practices, such as polygamy, that impeded civilizational progress, in part on the grounds that they held women back from developing roles outside procreation and sexual gratification, as explained by Fisher and Funke's article in this special issue. As Jones and Sequeira show, these racialized frameworks were simultaneously produced and expounded by sexologists around the globe. Rovel Sequeira's article reveals how in India, nationalist attempts to embrace liberal modernity found fertile material in the racist literary and scientific infrastructures of modern sexology, which they adapted to present caste-bound regulation of marriage as necessary to India's national progress. Similarly, Jones's article argues that at the heart of Mexican sexology was a racialized understanding of the post-revolutionary nation as underdeveloped and in need of modernization after centuries of colonialism and conflict; this modernization required the promotion of reproductive interventions that encouraged the realization of the *gran familia mexicana*. These contributions reveal the ways sexual deviance was presented as a threat to civilization while sexualities seen as essential to the health of the nation were promoted. In Mexico, as Jones demonstrates, a process of hetero-normativizing, rhetorically framed as ‘forging the fatherland’, allowed queer adults to be imprisoned or exiled to penal colonies or be subjected to social and educational interventions. Moreover, for some European sexologists, as Moore's article highlights, the very existence of sexual science, perceived as a European innovation, served to underpin claims about the superiority of European civilization. In dismissing medieval Islamicate sexual medicine as primitive, pornographic, and unscientific, European sexology presented itself as the singular product of a racially superior culture. Sexual knowledge itself was colonized and read as evidence of various cultures’ positions along civilizational trajectories. As Moore explains, colonized or subaltern forms of sexual knowledge were either ignored or seen as evidence of a ‘perverse, mysterious, exotic, and hypersexual Orient’.

At the root of sexology's commitment to imperial politics were evolutionary models of sexuality that saw the sexual instinct as the central mechanism through which societies developed. Fisher and Funke dissect the developmental dynamics behind the evolutionary understanding of the sexual impulse by sexual scientists such as Bloch, Ellis, Auguste Forel, Krafft-Ebing, and Moll. Seen as operating through desire as well as via reproduction, sexuality, they argued, drove moral, social, creative, and intellectual progress. Sexological knowledge thus positioned itself as of vital importance in the development of global racial health. It could identify the evolutionary benefits of the sexual impulse and advise which bodies and desires required regulation and control. In Mexico, for example, as Jones’s article shows, sexologists characterized the nation as developmentally impeded by its complex ethnic heritage, especially the legacy of colonial racial mixing (*mestizaje*). Sexual science informed a national project of renewal that promised to identify congenital markers of perversion that were ready to be activated via somatic changes or inculcated by a corrupt environment. It offered to replace ‘primitive’ sexual corruption found in urban slums (sexual excess, premature sexual experiences, prostitution, and pornography), ‘convert’ indigenous populations away from tradition, and improve the ‘race’ through correct modes of sexual citizenship. In India, as Sequeira demonstrates, phenomena such as child marriage were mobilized to charge Indian culture with hypersexuality and diagnose the nation as ‘backward’ and incapable of self-governance. Modernizing sexual scientists committed to Indian nation building, such as Narayan Sitaram Phadke, argued that future development could be secured by young Indian couples who had evolved away from the lustful and animal instincts of the past by rediscovering the customs of their Aryan heritage. These modern couples were now committed to ongoing evolutionary progress via caste-contiguous marriages. As Fisher and Funke show, sexologists in Britain, Germany, and America, including female scientists and campaigners such as Marie Stopes, Alice Stockham, and Helene Stöcker, drew upon evolutionary models to support various campaigns for sexual reform. Non-reproductive expressions of heterosexuality, particularly those that enabled pleasure without pregnancy, were advocated as providing the necessary evolutionary conditions for women to reach higher social, moral, and intellectual skills, which would in turn lead to a more advanced civilization. Changes to anti-homosexuality laws, too, were championed on the grounds that non-reproductive desire between men drove the development of social love and altruism beyond the confines of the biological family.

Through examining the concept of development, the articles in this special issue also highlight the racialized infrastructure that underpinned sexology. Together, they shed light on our understanding of the nature of sexology and its development as a field. More than a project to identify and label individualized sexual identities, sexology extended its ambition to understanding the relationship between the sexual impulse and human evolution. In so doing, it also had a broad conception of its scientific reach. By combining psychological and psychiatric knowledge with arguments and evidence drawn from anthropology, history, literature, and the arts, sexology sought to apply its knowledge to the governance of global populations, and in so doing fostered rich transnational exchanges worldwide.

This special issue thus contributes to ongoing discussions about the cross-disciplinary and cross-national development of sexual science as a field (Crozier and Bauer, 2017). Whether one sees sexology as fundamentally defined by medical and scientific disciplines that ranged from psychiatry and forensic medicine to the biological sciences and endocrinology, or as a broader interdisciplinary field that included literature, history, the social sciences, and law, the development of sexual science was a transnational and global project (Kahan and LaFleur, 2023). From its inception, sexual science responded to local concerns and had regional characteristics, yet sexologists also engaged with colleagues in other countries and continents. A growing body of vital scholarship has shown that sexological knowledge did not only move from Western Europe and North America to the rest of the world, but was exchanged in multi-directional ways. South and East Asia, Africa, and Latin America produced sexological knowledge that crossed continental boundaries and circulated globally through networks and circuits (Haynes, Fuechtner, and Jones, 2018; Stoler, 1995). The preoccupation with disciplinary boundaries characterizing much recent scholarship is not merely a historiographical debate. It was also shared by sexologists in the past, as the conflict between sexology and psychoanalysis at the beginning of the 20th century demonstrates (Sutton, 2019). Although the disciplinary boundaries between sexology and psychoanalysis were blurred, some sexologists, including Hirschfeld, distanced themselves from psychoanalysis. Moreover, some figures that we now identify as sexologists did not claim this role; in Italy, for instance, Cesare Lombroso preferred to refer to himself as a psychiatrist or criminal anthropologist (Beccalossi, 2012: 117–46; Sutton, 2019: 51–5).

Contention about the contours, boundaries, and authority of sexual science were fundamentally influenced by the tendency to think transnationally, to look outward, and to situate scientific knowledge within wider global and transnational networks, as several articles in this special issue demonstrate. Between the end of the 19th century and the beginning of the 20th century, a sense of professional marginalization encouraged a number of sexologists to engage with colleagues across national borders. The proliferation of various international networks and circuits of knowledge production increased over the course of the 20th century. As Jones's article shows, Mexican sexologists drew on and were in conversation with the Italian and Spanish endocrinologists Nicola Pende and Gregorio Marañón. In turn, Marañón developed his sexological work on ‘intersexual states’ in conversation with the Latvian-Chilean physiologist Alexander Lipschütz, who was based at the University of Concepción in Chile (MacMillan, 2018). In this context of field expansion and transnational exchange, development was a concept at stake in securing the authority and status of sexual science. Various sexologists delimited their understanding of the field, tracing the roots of sexual science in scientific traditions, foundational texts, and historical trajectories.

Debates about the boundaries and authority of sexual science were also influenced by comparative historical and ethnographic arguments. Classifying forms of knowledge as more or less ‘developed’, ‘progressive’, or ‘advanced’ played a constitutive role in the legitimation of the sexological project. Some sexual scientists presented their work as ‘modern’ and ‘new’, insisting that it signalled a radical break with the past; yet modern sexual science was also frequently seen to emerge from older forms of knowledge. As Moore’s article shows, in the context of Western sexology, the undervaluing of ancient and non-European forms of medical knowledge about sex, even one as substantial as earlier Islamicate sexual medicine, supported a particular origin story about sexology's own emergence and development as a supposedly new and unprecedented biomedical and scientific way of knowing. In addition, sexological knowledge drew on cross-historical and cross-cultural comparison to classify and judge different sexual behaviours. Earlier sexological studies focusing on so-called sexual psychopathologies were filled with historical references to a past that was presented as lascivious and permissive. Sexologists also attempted to draw parallels between new sexological categories and forms of behaviour found in past civilizations. For example, in *L’uomo delinquente* (Criminal Man), Lombroso linked modern manifestations of same-sex desires to the ‘pederasty’ practised in ancient Greece and Rome (Lombroso, 1876). In the first chapter of *Sexual Inversion*, Ellis and Symonds gave an overview of the prevalence of homosexual practices across history and across the globe (Ellis, 1915: 1–64).

The sexological tendency to think in ethnographic terms was fuelled by increasing knowledge of global diversity, which was a continuation of earlier world exploration embedded in processes of colonization and imperialism. As several scholars have shown, Western sexual science was shaped by preconceptions about Others considered to be positioned outside ‘modernity’ (Haynes, Fuechtner, and Jones, 2018). Howard Chiang (2009), for instance, refers to the ‘double alterity’ of sexology, arguing that the epistemic premise for the scientification of sexology is the convergence of geographical and temporal alterities. Western sexologists built a supposedly ‘modern’ sexual science on two premises: first, many non-Western societies were presented as ‘uncivilised’ and ‘savage’; second, past sexual customs reflected societies that were assumed to represent lower levels of evolutionary development. Sexological arguments, then, were often sustained by evolutionary models that imposed hierarchies upon different ethnic groups and tended to uphold fantasies of Western and white superiority. As Fisher and Funke's article suggests, British and German sexologists, for instance, sought to understand the social functions of the sexual instinct by mobilizing narratives of human evolutionary development that reflected racist and imperial ideologies. The increasing integration of anthropology, ethnology, and history into sexual science served different purposes, allowing, for example, for less medical and pathologizing views of sexuality to emerge (Funke and Fisher, 2018). This is not to say, however, that sexual science moved from a phase of pathologization towards one of liberation, a linear narrative supported by past sexologists and early historians of sexology. As all the articles included in this special issue emphasize, complex and multi-directional developments in the field persist on national, transnational, or global levels.

To provide a more detailed overview of the contributions to this special issue, Moore's article investigates the determined lack of interest in the *ʿilm al-bah* tradition of sexual medicine shown by French, English, German, and Austrian sexologists in the 19th and early 20th centuries. She argues that rather than engaging with these complex texts, sexologists dismissed the knowledge they represented as unscientific. In characterizing and labelling this literature as an ‘erotology’, sexual scientists categorized the form of knowledge it represented as primitive, pornographic, and itself a demonstration of Eastern perversion. Islamicate erotology was thus brought into existence in a form that served as a productive contrast with European sexology, whose scientific approach demonstrated the higher knowledge produced by a racially superior, developed, and progressive society. Moore contextualizes the success of the dismissive labelling of Islamicate erotology within pre-existing racial and colonial assumptions about the exotic excesses of Arabs and Muslims. Moreover, she suggests that an ongoing legacy of these prejudicial responses to Islamicate sexual medicine can be traced in the 20th- and 21st-century history of sexuality. Study of *ʿilm al-bah* texts remains underdeveloped, informed, she suggests, by an ongoing Eurocentrism in both contemporary sexual medicine and histories of sexuality, which continue to focus predominantly on the study of late 19th-century sexology rather than on earlier global traditions of sexual knowledge.

Fisher and Funke focus on exploring the ways in which Western European sexual scientists, especially in Britain and Germany, understood the sexual instinct, arguing that it provides a dynamic thread through which to chart the history and development of sexology. They argue that non-reproductive practices, such as homosexuality, masturbation, or non-reproductive forms of heterosexual desire, were not exclusively seen as perversions straying from a purely reproductive sexual impulse; nor were they necessarily viewed as primitive or atavistic. Instead, sexual scientists constructed the sexual instinct as an evolutionary force responsible for social, moral, and cultural evolution, both past and present, and sought to investigate the relationship between the myriad manifestations of human sexual desire and the trajectories of human civilizational progress. Fisher and Funke show how such explorations, in assessing the perceived evolutionary benefits or harms of various sexual behaviours and customs, drew upon the racialized, gendered, and classed hierarchies that distinguished between different cultures in the context of European imperial politics. Their article therefore demonstrates that investigating sexological theorizations of the sexual instinct can help to reveal ways in which sexual science was involved in imperial politics of social improvement; sexology offered the promise of a behavioural blueprint for the achievement of civilizational progress via the careful organization of sexual life.

Sequeira investigates the use of similar evolutionary frameworks, turning attention to the developmental temporalities structuring sexual scientific thought in colonial India. Sequeira's article focuses specifically on the work of sexological writer and novelist N. S. Phadke, who published in both Marathi and English. Phadke responded to the eugenicist implications of the Child Marriage Restraint Act of 1929 by presenting romantic love as a form of sociability that could drive social development and bring about a desired eugenic modernity. While recognizing existing laws as deficient in organizing sexual life, Phadke's work was nevertheless aligned with the state's biopolitical goals of governing modern coupledom. Sequeira's article also draws attention to entanglements between sexological and literary writings by studying Phadke's scientific treatises alongside his literary fiction. Phadke used the novel form, specifically the developmental narratives of the bildungsroman, to ensure that eugenic sexological framings of intimacy could be disseminated among ‘vernacular’ regional audiences in Marathi. In so doing, Phadke's work advocated caste-bound romance as key to the development of modern sexual life and democratic liberalism in interwar India.

Furthering this special issue's exploration of the intersections between sexology and eugenics, Beccalossi's article considers how sexologists who employed hormone research in Spain, Italy, Argentina, and Brazil interpreted human sexual development in the interwar period. Hormones came to be understood as the chemical messengers that regulated an individual's growth and sexual development. Beccalossi shows that the first stages of human life were fundamentally seen as sexually undifferentiated. Sexologists who worked at the intersection of endocrinology, eugenics, and biotypology focused on what they referred to as ‘intermediate conditions’ and acknowledged the existence of considerable variation in sex characteristics, challenging the binary understanding of sex. However, the fact that sexologists contemplated the richness of sexual variation did not mean they valued diversity. Beccalossi demonstrates that in Spain, Italy, Argentina, and Brazil, sexologists who employed hormone research promoted a specific understanding of the body and the sexual instinct, arguing that they were fundamentally malleable. As a result, hormone therapies were used to ‘correct’ and ‘normalise’ homosexuals and individuals with intersex variations.

The final article, by Jones, explores how Mexican sexology approached homosexuality and gender nonconformity from the late 1920s to the late 1950s. By focusing on case studies of ‘pederasts’, who appeared in specialized youth courts, and gender nonconforming people such as Marta Olmos Romero, who underwent Mexico's first widely known medical transition, Jones shows that Mexican sexologists viewed sexuality as caused mainly by social and environmental factors, rather than simply as a congenital characteristic. Mexican sexologists advocated for social solutions to the dangers posed by homosexuality and gender nonconformity, targeting their interventions towards youths, who were considered pliable future citizens, rather than adults, who were largely seen as already irredeemable. Jones demonstrates the important intersections between sexology, anthropology, eugenics, and biotypology, and the way in which the field of sexology became professionalized in Mexico. Finally, the article shows how the attempted management of homosexuality and gender nonconformity sheds light on the development of sexology in Mexico.

Individually and collectively, these articles reveal how the richly resonant concept of development can help to expand existing scholarship on the history of sexology. In different ways, the contributions to this special issue demonstrate the breadth of the sexological field in terms of its interdisciplinary scope, diverse political and intellectual agendas, and global dimensions. By exploring sexological projects in multiple locations across the globe, the authors engage with the various ways in which sexologists relied upon individual, national, and global developmental narratives that were frequently underpinned by ideologies of nation, race, and empire. We hope that this special issue will contribute to vibrant debates about the historical development of sexology while also encouraging readers to reflect upon the development of the history of sexology as a field of study.
